# Development of Biscuit Formulation Enriched With Tomato Paste Waste Using Integrated AHP‐TOPSIS Method

**DOI:** 10.1002/fsn3.70051

**Published:** 2025-02-19

**Authors:** Ezgi Ozgoren Capraz, Ozan Capraz, Figen Turan, Fatma Isik

**Affiliations:** ^1^ Department of Food Engineering Pamukkale University Denizli Türkiye; ^2^ Department of Industrial Engineering Pamukkale University Denizli Türkiye; ^3^ Department of Chemical Engineering Pamukkale University Denizli Türkiye

**Keywords:** AHP, biscuit, enrichment, tomato paste waste, TOPSIS

## Abstract

A high amount of waste is generated in tomato paste production. This waste contains tomato peel and seeds which are rich in food components such as carotenoids, phenolic compounds, mineral matters, dietary fibers, proteins, and fats. Therefore, it draws attention as a good enrichment material. In this study, biscuit production was carried out using tomato paste production waste powder (TPWP) at ratios of 0% (control), 6% (TPW6), 12% (TPW12), and 18% (TPW18). In the study, firstly, various chemical, physical, and sensory properties of the biscuit samples were determined. In biscuits, the amount of protein, fat, ash, total dietary fiber, insoluble dietary fiber, P, K, Ca, Mg, Mn, total phenolic, lycopene and β‐carotene contents, a* and b* color values, and antioxidant activity value increased significantly as the TPWP addition ratio increased (*p* < 0.05); carbohydrate ratio, energy value, and L* value decreased significantly (*p* < 0.05). In light of the results from sensory analysis, the samples produced by adding TPWP up to 12% had similar scores as the control sample in terms of color, odor, hardness, and chewiness parameters (*p* > 0.05). In addition, overall acceptability scores and taste decreased with the increase in the TPWP addition ratio. Finally, biscuit samples were examined in terms of functional (total dietary fiber, lycopene, β‐carotene, and antioxidant activity) and sensory properties (taste, color, chewiness) using the integrated AHP‐TOPSIS method, and the optimum product formulation was determined as TPW12 according to these criteria. Total dietary fiber, total phenolic contents and antioxidant activity of TPW12 were found to be approximately 2.2‐, 5.6‐ and 13.6‐ fold higher compared to the control sample, respectively. Additionally, lycopene and β‐carotene contents of TPW12 were 6.57 and 14.75 ppm, whereas no lycopene and β‐carotene were detected in the control sample. The results of the study revealed that TPWP could be an excellent source of dietary fiber, lycopene and β‐carotene for biscuits.

## Graphical abstract

In the “State of Food and Agriculture 2019” report of FAO, it was stated that reducing food waste will contribute to food security and sustainability goals globally. In this study, the aim was to utilize tomato paste production waste in biscuits from the perspective of sustainability. In addition, the use of the integrated AHP‐TOPSIS method in the selection of the optimum biscuit formulation would provide an innovative way for other researchers working in this field.

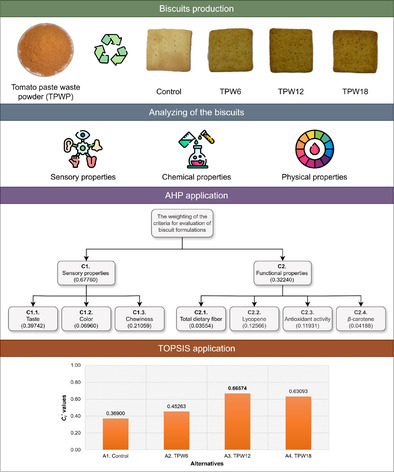

## 1 Introduction

According to the data from the Food and Agriculture Organization (FAO), approximately 189 million tons of tomatoes were produced in the world in 2021. China, India, Turkey, the USA, Italy, Egypt, and Iran are the leading tomato producer countries (FAO 2022; TEPGE 2022). A significant proportion of the tomatoes produced are processed into tomato products such as ketchup, puree, tomato paste, tomato juice, and canned food (Isik and Topkaya 2016). The industrial processing of tomatoes results in a significant amount of waste in addition to the final processed product. In a world where environmental pollution is rapidly increasing, one of the most important efforts is the treatment or valorization of wastes without causing environmental pollution. The valorization process can be considered as the process of transforming waste into other value‐added products. In the production of such products, priority should be given to those, such as cereal, dairy, and meat products, that play an essential role in daily nutrition.

In tomato paste production, 10%–30% of the raw material is separated as pulp which consists mainly of peel and seeds (Ghazi and Drakhshan 2006). This pulp is often sold as animal feed for very low prices or used as fertilizer (Knoblich et al. 2005). In some studies, it has been emphasized that biologically active compounds including carotenoids, especially lycopene and β‐carotene, phenolic compounds, mineral matters, dietary fibers, proteins, and fats are abundantly found in tomato pulp (Calvo et al. 2008; Isik and Yapar 2017; Knoblich et al. 2005; Persia et al. 2003). Therefore, it is necessary to consider alternatives to offer such a nutrient‐rich material for human consumption. In the literature, it is seen that among the food groups, bakery products are in the first place in enrichment studies. Biscuits are one of the most consumed bakery products as they are ready‐to‐eat food, are nutritious and satisfying, can be produced in various flavors, and are economical and appealing to a wide range of consumers (Demirel and Demir 2018; Urganci and Isik 2021).

This study aimed to analyze the biscuit samples produced with the addition of tomato paste production waste powder (TPWP) at various ratios using the integrated Analytic Hierarchy Process (AHP) and Technique for Order Preference by Similarity to Ideal Solution (TOPSIS) approach in terms of functional and sensory properties and to select the optimum formulation.

Decision‐making can be defined as the process of selecting, ranking, or categorizing among available alternatives to address a given problem. On the other hand, multi‐criteria decision‐making involves the consideration of multiple and often conflicting criteria. One such method, TOPSIS enables the ranking of alternatives by evaluating their proximity to the positive ideal solution and their distance from the negative ideal solution. The TOPSIS method is well‐known in the literature for its computational simplicity, flexibility, and its ability to evaluate multiple criteria simultaneously, rather than focusing on a single criterion. Consequently, the TOPSIS method has been proven to be an effective tool in decision‐making processes involving a range of criteria. Given these advantages, the method has found widespread application across various fields including the food sector (Alan et al. 2023; Miç and Antmen 2021).

There are examples in the literature where the TOPSIS method has been successfully applied in areas such as optimization of production parameters and development of product (juices, breads, cakes, etc.) formulation. For example, Gul et al. (2018) and Agustina et al. (2020) used TOPSIS as an effective tool for determining the optimum conditions in the production of various products, meanwhile Ozturk et al. (2014), Yılmaz and Koca (2020) and Hedayati et al. (2022) demonstrated the potential of this method in the development and selection of product formulations.

In the study, instead of selecting the best formulation objectively based on a single criterion such as overall acceptability score, an integrated AHP‐TOPSIS multi‐criteria decision‐making approach was used considering several functional and sensory properties. In the “State of Food and Agriculture 2019” report of FAO, it was stated that reducing food waste will contribute to food security and sustainability goals globally (FAO 2019). In this study, the aim was to utilize tomato paste production waste (TPW) in biscuits from the perspective of sustainability. In addition, the use of the integrated AHP‐TOPSIS method in the selection of the optimum biscuit formulation would provide an innovative way for other researchers working in this field.

## 2 Materials and Methods

### 2.1 Materials

Wheat flour ( *Triticum aestivum* ), margarine, baking powder, salt, and sugar were obtained from local markets in Denizli, TPW was supplied from Honaz Salça Factory (Honaz/Denizli).

**FIGURE 1:**
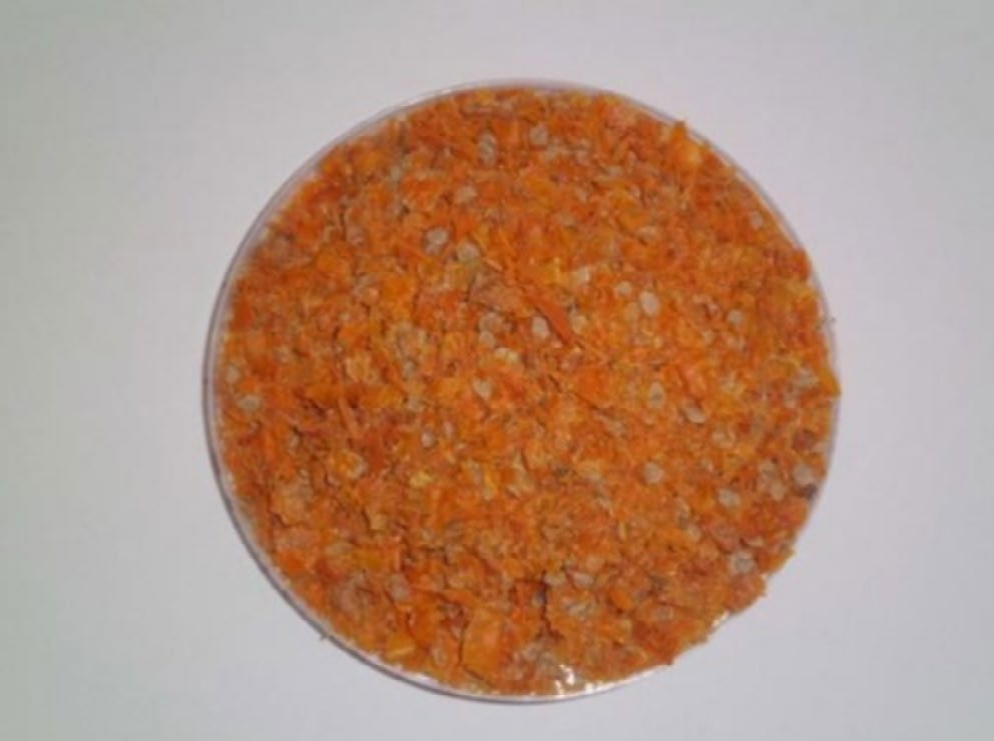
Tomato paste waste.

**FIGURE 2:**
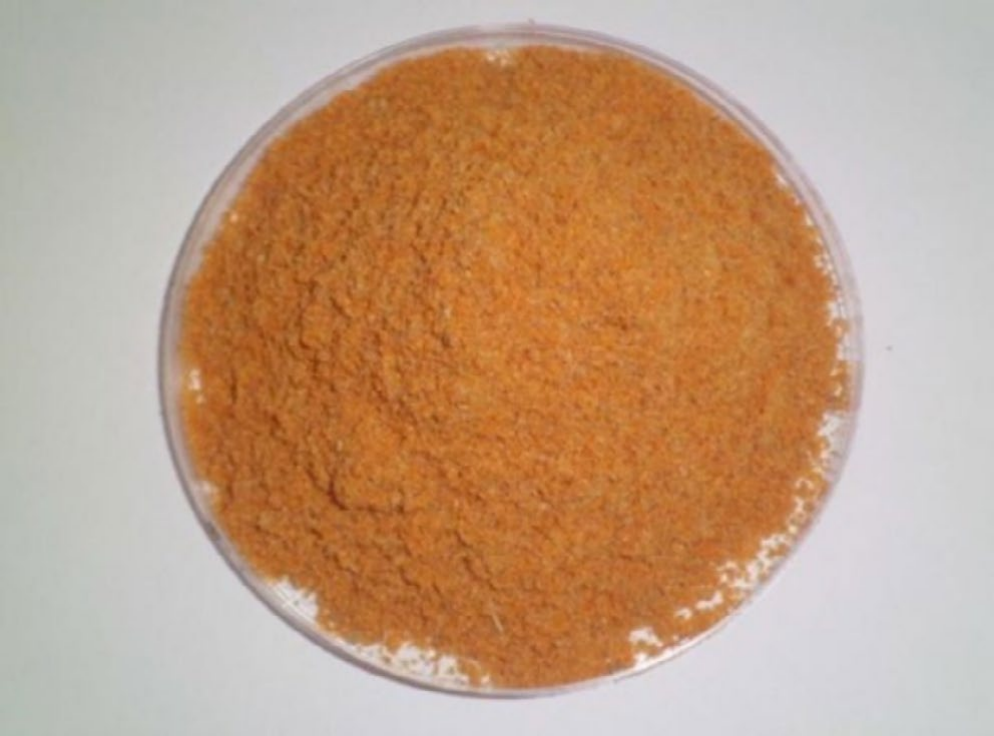
Tomato paste waste powder.

TPW was dried at 60°C in an air‐flow dryer (Yücebaş Machine, İzmir/Türkiye) until the moisture content decreased below 10% (Figure 1) and then ground (Toper TKS‐16S, İzmir/Türkiye) to a particle size of < 500 μm (Figure 2).

### 2.2 Methods

**FIGURE 3:**
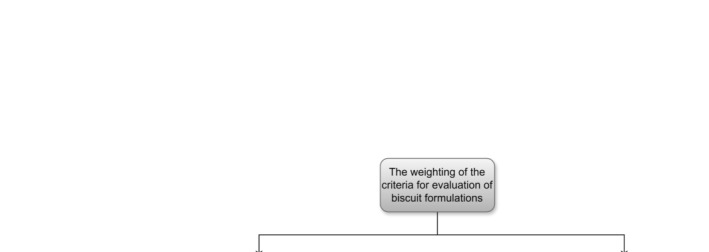
Hierarchical structure of the AHP method.

In the study, firstly, control biscuits and enriched biscuits that wheat flour substituted with TPWP at ratios of 6% (TPW6), 12% (TPW12), and 18% (TPW18) were produced, and then some sensory, physical, and chemical properties of the biscuits were determined. Later, the integrated AHP‐TOPSIS method was employed to determine the optimum biscuit formulation. Nine academicians from Pamukkale University, Department of Food Engineering were involved as experts in the application of the method. Within the scope of the research, the AHP and TOPSIS methods were introduced to the experts. The experts then determined the criteria to be used in selecting the optimum biscuit formulation by considering the sensory and functional properties of the biscuits. The criteria for selecting the optimum biscuit formulation based on the experts' opinions were organized in a hierarchy as shown in Figure 3.

Within this hierarchy, there were two main criteria, namely sensory properties (C1) and functional properties (C2). Each main criterion was divided into sub‐criteria. There were three important sub‐criteria under sensory properties: taste (C1.1), color (C1.2), and chewiness (C1.3). There were four important sub‐criteria under functional properties: total dietary fiber (C2.1), lycopene (C2.2), antioxidant activity (C2.3), and β‐carotene (C2.4). All sub‐criteria were identified as maximization criteria where higher values were preferred.

**FIGURE 4:**
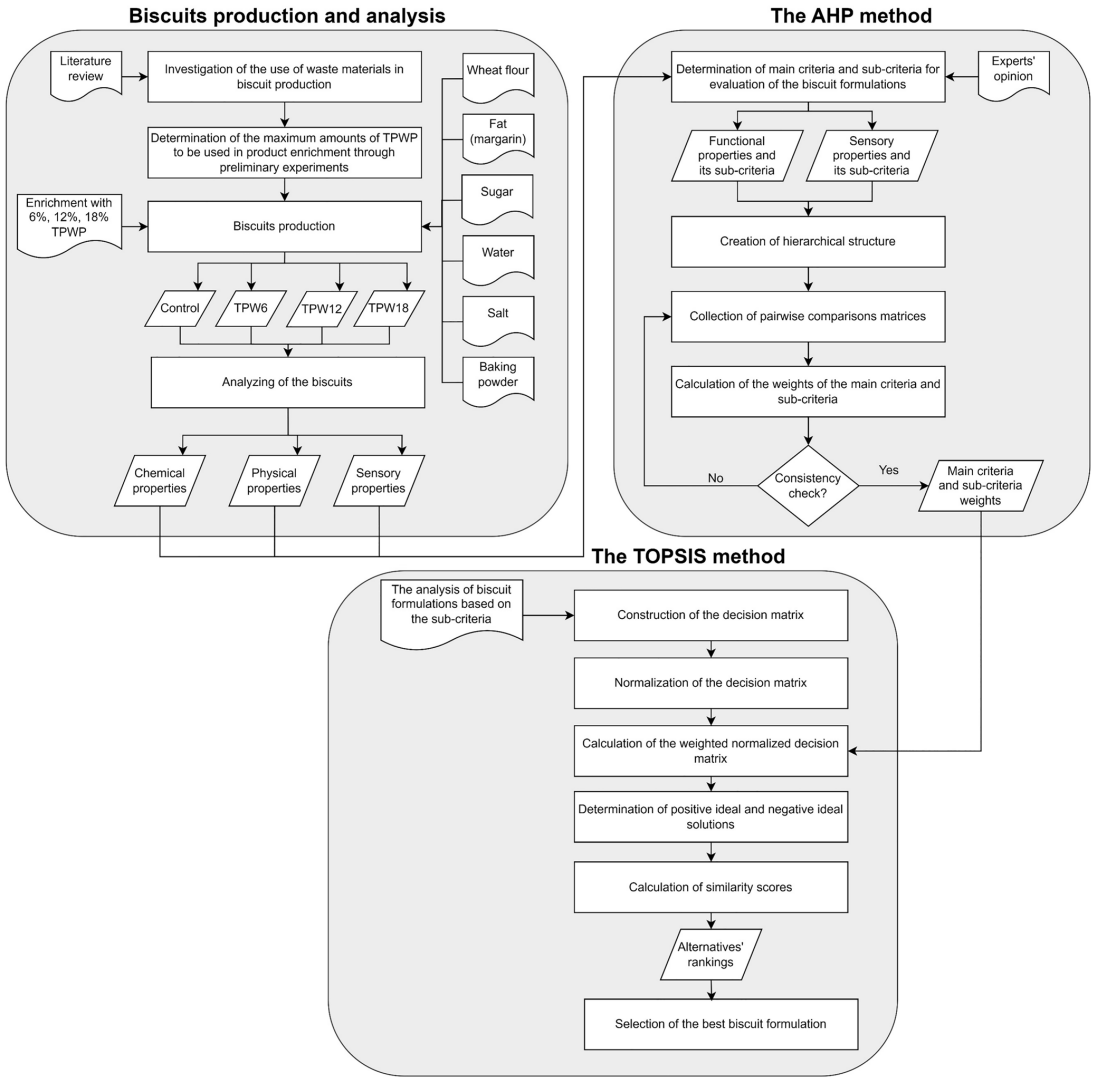
The methodological framework of the study.

The methodological framework followed in the study is presented in Figure 4.

While the AHP method was used to determine the weights of the criteria, the TOPSIS method was used to rank the alternatives according to these criteria. This study reveals the applicability and effectiveness of these methods for decision‐making problems encountered in the development of new products. In the AHP method, the pairwise comparison matrices between the criteria were collected individually from the experts, and the consistency rates were checked. To ensure reliability in the AHP method, consistency ratios were evaluated for each matrix. The matrices with a consistency ratio above 0.1 were re‐consulted with the relevant experts and these matrices were revised. Nine pairwise comparison matrices were combined using the geometric mean method (Aczél and Saaty 1983). The resulting pairwise comparison matrix was used in the AHP calculations. A reliable and effective software, SuperDecisions V3.2 (Creative Decisions Foundation, USA) was preferred in the process of applying the AHP method. Considering the assessment criteria, the results obtained from the sensory and functional analyses of the biscuits were used for the evaluation scores of the alternative biscuit formulations. After applying the steps of the TOPSIS method, the alternatives were listed, and the optimum biscuit formulation was determined.

#### 2.2.1 Biscuit Production

Biscuits having TPWP were prepared by substituting 6%, 12%, and 18% of the wheat flour with TPWP. Biscuit formulations are seen in Table 1.

**TABLE 1:** Formulations of biscuits.

Ingredients (g)	Control	TPW6	TPW12	TPW18
Wheat flour	100.0	94.0	88.0	82.0
TPWP	0.00	6.0	12.0	18.0
Fat (margarin)	40.0	40.0	40.0	40.0
Sugar	35.0	35.0	35.0	35.0
Water	20.0	20.0	22.0	24.0
Salt	0.7	0.7	0.7	0.7
Baking powder	0.5	0.5	0.5	0.5

In biscuit production, all ingredients (Table 1) were poured into the kneading bowl of a table mixer (KMM060 Kitchen Chef, Kenwood) and were kneaded for 5 min until the dough became homogeneous. The dough was rested for 10 min and then thinned down to 2 mm by passing through the dough sheeter of the table mixer (KitchenAid, Model 5KSM150, USA). It was then cut into 5 cm x 5 cm pieces with a biscuit mold. The biscuit doughs were baked in an electric fan oven at 200°C for 10 min.

#### 2.2.2 Proximate Composition Analysis

Moisture (method 934.01), protein (method 988.05), ash (method 942.05), and fat (method 954.02) contents were determined according to the methods described by AOAC (2005a). Dietary fiber analysis was performed according to AOAC (2005b) 991.43 and AACC (2000) 32–07 methods using a total dietary fiber analysis kit (Megazyme K‐TDFR, Wicklow, Ireland). Carbohydrate content (%) was computed by subtracting ash, protein, moisture, fat, and dietary fiber from 100 (Abd Rabou 2018). The energy value was computed by multiplying the fat amount by 9 kcal/g, protein and carbohydrate amounts by 4 kcal/g, and summing these values. Energy value results are given in kcal/100 g (Souci et al. 2000).

#### 2.2.3 Total Phenolic Content and Antioxidant Activity

Phenolic compounds were extracted according to the method described by Ozgoren et al. (2019). Total phenolic content analysis was performed following the Folin–Ciocalteu (FC) method (Singleton et al. 1999) and results are given as mg gallic acid equivalent (GAE)/100 g. Antioxidant activity values were determined using the DPPH method (Thaipong et al. 2006) and results are given as μmol Trolox equivalent (TE)/100 g.

#### 2.2.4 Lycopene and β‐Carotene Content

For the carotenoids extraction, 4 g of sample was homogenized for 1 min in 40 mL ethanol: hexane (4:3) mixture containing 1% (w/v) butylated hydroxytoluene. After homogenization, the samples were centrifuged (NF 1200 R, Nüve, Türkiye) at 9000 *g* for 15 min at 4°C and the supernatants were collected in amber bottles. Before injection into HPLC device (Thermo‐fischer, USA), supernatants were filtered through 0.45 μm membrane filters (Tumer and Tulek 2023).

An acetonitrile:methanol:dichloromethane:hexane mixture (40:20:20:20, v/v/v/v) was used as a mobile phase in HPLC. The flow rate of the mobile phase was 0.45 mL/min at 25°C and the analysis time was 20 min. The detection wavelength was 470 nm for lycopene and 445 nm for β‐Carotene. The amounts of lycopene and β‐carotene were calculated based on the standard curve (Tümer et al. 2023). Results are given in ppm.

#### 2.2.5 Mineral Matter Composition

In mineral matter analysis, ICP‐OES (Perkin Elmer, Optima 8000 model, Massachusetts, USA) was used to determine the amounts of P, K, Ca, Mg, and Mn in the samples. Results are given in ppm (Boss and Fredeen 2004).

#### 2.2.6 Color Analysis

The color values of the raw materials and biscuit samples were determined according to the CIE color system (L*, a*, b*) using a Hunter‐Lab Mini Scan XE colorimeter (Hunter Associates Laboratory, Reston, USA). Moreover, ∆*E* values were calculated using Equation (1) to determine the total color change between the control and enriched samples.

$$∆E=\sqrt{{\left(∆L\right)}^{2}+{\left(∆a\right)}^{2}+{\left(∆b\right)}^{2}}$$

$$∆L={L^{*}}_{\text{sample}}-{L^{*}}_{\text{control}}$$

$$∆a={a^{*}}_{\text{sample}}-{a^{*}}_{\text{control}}$$

(1) $$∆b={b^{*}}_{\text{sample}}-{b^{*}}_{\text{control}}$$

According to the classification made by Yamauchi (1989), the visual color differences of total color change values (∆*E*) were classified as follows: larger than 12, another color group; 6.0–12.0, large difference in the same color group; 3.0–6.0, detectable by ordinary people; 1.5–3.0, detectable by trained people; 0.5–1.5, hard to detect with the human eye; 0–0.5, trace difference.

#### 2.2.7 Sensory Analysis

A total of 50 semi‐trained panelists (15 men, 35 women) from Pamukkale University participated in the sensory analysis. The biscuits were evaluated for color, odor, hardness, taste, chewiness, and overall‐acceptability using a 7‐point hedonic scale. Water was given to the panelists between tasting each sample for rinsing their palates. All participants were informed and provided written consent prior to their involvement in the sensory analysis.

#### 2.2.8 AHP Method

In the literature, the AHP method is preferred in many fields due to its advantages such as the inclusion of personal evaluations (preferences) of decision‐makers in the problem‐solving, allowing the measurement of consistency in pairwise comparison matrices containing personal evaluations, allowing the evaluation of multiple qualitative and/or quantitative criteria, sub‐criteria, or lower level criteria, and allowing individual or group decision‐making (Saaty 1987, 1988). The steps of the AHP method are summarized below (Saaty and Vargas 2012; Xia and Wu 2007):

1. Identification of the problem and establishment of the hierarchical structure,
2. Construction of pairwise comparison matrices,
3. Normalization of pairwise comparison matrices,
4. Calculation of priority vector,
5. Calculation of consistency ratio (CR),
6. Obtaining the overall result of the hierarchical structure.

#### 2.2.9 TOPSIS Method

The TOPSIS method is one of the most widely used multi‐criteria decision‐making methods for selecting the most appropriate alternative among all by considering multiple criteria (Hwang and Yoon 1981). Based on the evaluations of decision makers, in the TOPSIS method, the alternatives are ranked by evaluating both the proximity to the positive ideal solution and the distance from the negative ideal solution simultaneously. The TOPSIS method requires the weights of each criterion and the performance values of each alternative for each criterion (Chen and Tzeng 2004). The steps of the TOPSIS method are summarized below (Hwang and Yoon 1981; Wang et al. 2017):

1. Construction of the decision matrix,
2. Calculation of the normalized decision matrix,
3. Calculation of a weighted normalized decision matrix,
4. Determination of positive ($A^{*}$) and negative $\left(A^{-}\right)$ ideal solutions,
5. Calculation of distances of alternatives to positive ideal solution $\left(S_{i}^{*}\right)$ and distances of alternatives to negative ideal solution $\left(S_{i}^{-}\right)$,
6. Calculation of the closeness coefficient $\left(C_{i}^{*}\right)$.

#### 2.2.10 Statistical Analysis

All statistical analyses were performed using SPSS 22 (IBM Corp, Armonk, NY, USA). The results were compared using the Duncan multiple comparison model with a confidence interval of *α* = 0.05. All tests were performed at least in triplicate.

## 3 Results and Discussion

### 3.1 Chemical Compositions of Raw Materials

Table 2 shows some chemical properties and color values of wheat flour and TPWP. As seen in the table, the protein, fat, soluble, insoluble, and total dietary fiber, P, K, Ca, Mg, and Mn ratios of TPWP were higher compared to wheat flour whereas the carbohydrate and energy values were lower. The results found for the chemical compositions of TPWP and wheat flour are consistent with the literature (Alvarado et al. 2001; Del Valle et al. 2006; Isik and Topkaya 2016; Isik 2013) and it is thought that the small differences are due to factors such as ecological conditions, species, maturity period, and agricultural practices (Toledo and Burlingame 2006).

**TABLE 2:** Chemical compositions and color values of wheat flour and TPWP.

Parameters	Wheat flour	TPWP
Moisture (%)	10.35	6.87
Protein (%)	9.73	15.17
Ash (%)	0.425	3.238
Fat (%)	1.30	9.75
IDF (%)	1.36	47.70
SDF (%)	1.25	5.73
TDF (%)	2.61	53.43
Carbohydrate (%)	75.59	11.54
Energy value (kcal/100 g)	352.98	194.59
Total phenolic content (mg GAE/ 100 g)	98.68 ± 6.10	431.63 ± 27.02
Antioxidant activity (μmol TE/ 100 g)	2.15 ± 0.60	88.78 ± 4.86
β‐Carotene (ppm)	ND	50.51 ± 1.45
Lycopene (ppm)	ND	168.10 ± 6.83
P (ppm)	1444.25 ± 52.11	4588.25 ± 52.11
K(ppm)	1935.50 ± 21.07	24845.05 ± 487.55
Ca (ppm)	387.50 ± 10.61	3571.00 ± 77.07
Mg (ppm)	414.45 ± 22.84	2895.35 ± 63.29
Mn (ppm)	9.30 ± 0.28	39.05 ± 1.48
Hunter color values		
L*	94.35	55.23
a*	0.43	15.80
b*	9.17	19.67

In the study, total phenolic content and antioxidant activity value of TPWP were higher than wheat flour. In addition, TPWP contained 168.10 ppm lycopene and 50.51 ppm β‐carotene while lycopene and β‐carotene were not detected in wheat flour. In the literature (Baysal et al. 2000; Sikora et al. 2008; Verhoeyen et al. 2002), it has been reported that the peels and seeds, which constitute the main part of TPWP, contain carotenoids such as lycopene and β‐carotene and polyphenolic compounds such as quercetin, rutin, campferol, and chlorogenic acid in considerable amounts. Therefore, it was expected that the total phenolic content and antioxidant activity values of TPWP were higher than wheat flour. In the literature, it was observed that tomato peel dried under different conditions contained 167.4–734.0 μg/g dw lycopene and 28.9–55.2 μg/g dw β‐carotene, while tomato seeds contained 16.0–167.0 μg/g dw lycopene and 0.9–55.0 μg/g dw β‐carotene (Albanese et al. 2014; Knoblich et al. 2005; Kumar et al. 2021). Considering that the peel and seeds constitute the main part of TPWP, the findings of the current study are consistent with the literature.

In the study, it was also found that the color of TPWP was darker than wheat flour and that the redness and yellowness values were higher than that of wheat flour. This result is consistent with the findings in the literature (Bhat and Ahsan 2015; Isik 2013; Isik and Topkaya 2016). Carotenoids, which are abundant in tomato pulp, are fat‐soluble pigments that give fruits and vegetables a yellow to red color: lycopene is responsible for the red, β‐carotene for the yellow‐orange color (Maoka 2020; Rodriguez‐Concepcion et al. 2018). It is thought that the higher redness (a*) and yellowness (b*) values of TPWP compared to wheat flour are related to the lycopene, β‐carotene, and other carotenoids it contains.

### 3.2 Chemical Compositions of Biscuits

Table 3 shows the chemical composition of the biscuit samples. It was found that protein, fat, ash, insoluble dietary fiber, and total dietary fiber ratios of the biscuits increased significantly (*p* < 0.05) and the carbohydrate ratio and energy value of the biscuits decreased significantly (*p* < 0.05) as the TPWP added to the biscuits increased. The addition of TPWP to the biscuits did not cause a significant change in the soluble dietary fiber ratios of the samples (*p* > 0.05). These findings are associated with the differences between the compositions of TPWP and wheat flour used in biscuit production (Table 2). The increase in the substitution rate of TPWP, which has higher protein, fat, ash, and dietary fiber content, to wheat flour led to an increase in the values of these properties in biscuits. Likewise, as the rate of TPWP substitution to wheat flour increased in tarhana production in the study of Isik (2013) and cracker production in the study of Isik and Topkaya (2016), ash, dietary fiber, fat, and protein ratios of the products increased.

**TABLE 3:** Chemical compositions of biscuit samples.

Parameters	Biscuit samples			
	Control	TPW6	TPW12	TPW18
Moisture (%)	3.47 ± 0.18^a^	3.60 ± 0.01^a^	3.73 ± 0.10^a^	3.54 ± 0.11^a^
Protein (%)	5.95 ± 0.02^d^	6.16 ± 0.04^c^	6.37 ± 0.09^b^	6.53 ± 0.01^a^
Ash (%)	0.78 ± 0.03^b^	0.92 ± 0.01^ab^	1.00 ± 0.05^a^	1.08 ± 0.11^a^
Fat (%)	20.30 ± 0.12^c^	20.47 ± 0.08^c^	20.87 ± 0.08^b^	21.30 ± 0.03^a^
IDF (%)	1.08 ± 0.07^d^	3.19 ± 0.11^c^	4.69 ± 0.14^b^	7.10 ± 0.11^a^
SDF (%)	2.25 ± 0.21^a^	2.35 ± 0.35^a^	2.68 ± 0.07^a^	2.85 ± 0.04^a^
TDF (%)	3.33 ± 0.28^d^	5.54 ± 0.25^c^	7.37 ± 0.07^b^	9.94 ± 0.14^a^
Carbohydrate (%)	66.18 ± 0.01^a^	63.33 ± 0.28^b^	60.68 ± 0.11^c^	57.62 ± 0.18^d^
Energy Value (kcal/100 g)	471.16 ± 1.11^a^	462.15 ± 0.60^b^	455.95 ± 0.09^c^	448.28 ± 0.40^d^

*Note:* Different superscript letters in rows denote statistical differences (*p* < 0.05).

Food proteins are key ingredients for the formation of body proteins (Baysal 2022). Amino acids are the building blocks of proteins. After the proteins ingested with food are broken down in the human body and amino acids are released, the proteins necessary for the organism are synthesized from these amino acids (Metin 2001). Therefore, humans constantly need proteins, which are a source of amino acids. In the present study, substitution of wheat flour with TPWP at ratios of 6%, 12%, and 18% increased the protein content of the products by 3.53%, 7.06%, and 9.75%, respectively, compared to the control sample. The recommended protein intake is 50 g/day for adults (FDA 2023). It can be stated that the consumption of biscuits including TPWP would be a good alternative to biscuits produced only with wheat flour to meet the protein needs of individuals.

The recommended amount of dietary fiber to be taken daily is in the range of 20–35 g for adults (Marlett et al. 2002). According to the legislation, 1 serving of biscuit is 30 g (GTHB 2017). Based on the calculations from the dietary fiber content results in Tables 3, 1.0 g dietary fiber can be taken with the consumption of 1 serving of control group biscuit, while 1.66, 2.21, and 2.98 g dietary fiber can be taken with the consumption of 1 serving of TPW6, TPW12, and TPW18 biscuits, respectively. Therefore, the use of TPWP in biscuit production can significantly improve the dietary fiber content of the products.

A beneficial dietary model is associated with several factors, such as higher levels of dietary fiber, micronutrients, polyphenols, and unsaturated fatty acids; a lower glycemic index, salt, trans‐fat and refined carbohydrates. Moreover, the rapid digestion of low‐fiber carbohydrates (with a high glycemic index) has been demonstrated to drive many obesogenic pathways, which may result in an increased risk of cardiometabolic disease (Brand‐Miller et al. 2009; Mozaffarian 2016; Mozaffarian et al. 2013).

The American Heart Association's 2020 Strategic Impact Goals Committee has defined a ratio of total carbohydrate to dietary fiber (g/serving) of < 10:1 as a means of identifying grain products that offer a greater health benefit (Lloyd‐Jones et al. 2010; Mozaffarian et al. 2013). The carbohydrate: dietary fiber ratios of the TPW12 (9.23) and TPW18 (6.80) samples are both calculated as lower than 10 and meet the carbohydrate: dietary fiber ratio recommended by the American Heart Association's 2020 Strategic Impact Goals Committee.

Table 4 shows the total phenolic content and antioxidant activity values of the biscuit samples. As the TPWP substitution ratio increased, the total phenolic content and antioxidant activity values of the biscuits increased. Compared to the control sample, the total phenolic content of TPW18 sample increased approximately 6 times and the antioxidant activity value increased approximately 17 times. It can be stated that this finding is associated with the higher total phenolic content and antioxidant activity value of TPWP in comparison to wheat flour (Table 2).

**TABLE 4:** Total phenolic content and antioxidant activity of biscuit samples.

Parameters	Biscuit samples			
	Control	TPW6	TPW12	TPW18
Total Phenolic Content (mg GAE/ 100 g)	28.05 ± 2.69^d^	104.11 ± 6.05^c^	158.18 ± 4.21^b^	178.16 ± 2.86^a^
Antioxidant Activity (μmol TE/ 100 g)	2.08 ± 0.18^d^	10.42 ± 1.46^c^	28.23 ± 2.19^b^	35.47 ± 0.73^a^

*Note:* Different superscript letters in rows denote statistical differences (*p* < 0.05).

Table 5 shows the lycopene and β‐carotene amounts of the biscuits. There were no lycopene and β‐carotene detected in the control sample, while it was determined that the amounts of lycopene and β‐carotene increased according to the ratio of TPWP addition. While lycopene was about 3 times higher in TPWP compared to β‐carotene (Table 2), β‐carotene was higher in TPWP‐enriched biscuits. This was attributed to the fact that the biscuits were a product baked at 200°C and lycopene is more sensitive to high temperatures than β‐carotene (Sevindik Baç et al. 2023).

**TABLE 5:** Lycopene and β‐carotene content of biscuit samples.

Parameters	Biscuit samples			
	Control	TPW6	TPW12	TPW18
Lycopene (ppm)	ND	3.68 ± 0.15^c^	6.57 ± 0.13^b^	8.28 ± 0.19^a^
β‐carotene (ppm)	ND	12.85 ± 0.05^c^	14.75 ± 0.08^b^	16.75 ± 0.16^a^

*Note:* Different superscript letters in rows denote statistical differences (*p* < 0.05).

It is known that carotenoids are important sources of antioxidants. It has been reported that lycopene shows 2 times more antioxidant activity than β‐carotene. With these properties, lycopene and β‐carotene have many health benefits. It has been reported that consuming foods with high lycopene content reduces the risk of developing some types of cancer such as prostate cancer and epithelial cancer, cardiovascular diseases, and cataract disease (Kumcuoglu et al. 2014). β‐carotene has been reported to show high amounts of provitamin A activity (Gul et al. 2015). In light of these findings, it can be said that the bioavailability of biscuits will be increased with the addition of TPWP.

In the study of Kadam and Mane (2022), cookies were produced by substituting tomato pomace powder up to 16.67% in whole wheat‐millet‐oat flour mixture and it was found that the lycopene and β‐carotene contents of the cookies increased as the substitution rate increased. Similar to the present study, Kadam and Mane (2022) did not detect lycopene and β‐carotene in control samples.

In another study (Padalino et al. 2017), pasta was enriched with tomato peel flour at ratios of 10% and 15%. It was determined that the amounts of lycopene and β‐carotene increased approximately 35‐fold and 3‐fold, respectively, in the sample containing 15% tomato peel flour compared to the control sample.

According to Table 6, it was observed that there were significant increases (*p* < 0.05) in the P, K, Ca, Mg, and Mn ratios of the biscuits as the TPWP ratio used in the biscuits increased. It can be stated that the increase in mineral content is associated with the fact that TPWP contains these minerals significantly higher compared to wheat flour (Table 2). Similar increases were observed in the studies conducted by Isik (2013), Isik and Topkaya (2016), Mehta et al. (2018), Alazb et al. (2021), and El‐Araby (2022).

**TABLE 6:** Mineral matter compositions of biscuit samples (ppm).

Parameters	Biscuit samples			
	Control	TPW6	TPW12	TPW18
P	1375.45 ± 85.14^c^	1629.01 ± 64.74^b^	1702.76 ± 121.47^ab^	1901.89 ± 68.53^a^
K	1301.19 ± 117.32^d^	2199.00 ± 69.16^c^	2504.50 ± 23.33^b^	2997.00 ± 123.04^a^
Ca	948.09 ± 45.91^b^	1227.50 ± 168.62^a^	1322.98 ± 56.96^a^	1451.02 ± 51.39^a^
Mg	326.49 ± 32.75^c^	477.49 ± 29.30^b^	570.21 ± 37.89^ab^	651.44 ± 39.76^a^
Mn	6.91 ± 0.16^c^	8.50 ± 0.45^b^	8.72 ± 0.38^b^	9.77 ± 0.37^a^

*Note:* Different superscript letters in rows denote statistical differences (*p* < 0.05).

Minerals are essential elements of nutritional physiology that are involved in the structure and function of the body and cannot be synthesized by the organism. For example, Ca, P, and Mg are essential for the formation and maintenance of bones. Ca is also needed for the health of the heart, muscles, and digestive system. Mg and P are important for energy and ATP metabolism. K is a systemic electrolyte and is also required together with Na in the regulation of ATP. Mn is a cofactor in enzyme functions. Minerals are taken into the body through the diet and if the required amount is not taken into the body over a while, health problems such as goiter and anemia may occur (Godswill et al. 2020; Saldamlı and Sağlam 2007). In the literature, it has been reported that the daily Ca, P, K, Mg, and Mn intake needs of adults are 1000 mg, 800 mg, 2000 mg, 370 mg, and 2.0 mg, respectively (Baysal 2022; Saldamlı and Sağlam 2007). According to the calculations, an adult consuming 1 serving of control biscuits could meet 2.84%, 5.16%, 2.06%, 2.65%, and 10.5% of daily Ca, P, K, Mg, and Mn needs, respectively, whereas an adult consuming 1 serving of TPW18 could meet 4.35%, 7.13%, 4.50%, 5.28%, and 14.5% of their mineral needs, respectively.

### 3.3 Color Analysis

Table 7 shows the color analysis results of the samples. It was determined that the L* value decreased, and a* and b* values increased as the ratio of TPWP addition to the biscuit formulation increased. It was thought that the color difference in the samples was related to the original color of the wheat flour and TPWP (Table 2). In a study in which crackers were enriched with 4%, 8%, and 12% tomato pomace, it was determined that the *L* value decreased, and *a* and *b* values increased as the ratio of tomato pomace substitution in the formulation increased (Isik and Topkaya 2016). In another study, 4%, 6%, 8%, and 10% tomato pomace was used in the production of cream crackers. It was found that as the tomato pomace ratio increased in the formulation, the L* value decreased while the a* and b* values increased (Nakov et al. 2022). The color findings of the present study were found to be consistent with the literature.

**TABLE 7:** Color properties of biscuit samples.

Parameters	Biscuit samples			
	Control	TPW6	TPW12	TPW18
L*	76.92 ± 1.35^a^	69.08 ± 0.62^b^	65.97 ± 1.43^c^	61.93 ± 0.72^d^
a*	11.70 ± 0.59^b^	13.74 ± 1.36^ab^	14.60 ± 0.46^a^	15.93 ± 0.18^a^
b*	25.29 ± 0.21^d^	27.72 ± 0.69^c^	29.15 ± 0.06^b^	30.78 ± 0.33^a^
∆*E*		8.46 ± 2.16^b^	11.99 ± 0.40^b^	16.52 ± 0.53^a^

*Note:* Different superscript letters in rows denote statistical differences (*p* < 0.05).

The samples were also examined in terms of total color changes. According to the classification made by Yamauchi (1989), the TPW6 and TPW12 samples were classified as “large difference in the same color group” since their ∆*E* values were between 6.0–12.0, and the TPW18 sample was classified as “another color group” since its ∆*E* value was more than 12.

### 3.4 Sensory Analysis

Sensory analysis is one of the most important parameters determining consumer preferences. Table 8 shows the results of sensory analysis of biscuit samples. It was determined that the samples with TPWP added up to 12% had similar scores (*p* > 0.05) with the control sample in terms of color, odor, hardness, and chewiness parameters. In addition, taste and total acceptability scores decreased as the TPWP addition ratio increased. However, it was observed that all sensory quality parameters of the samples up to 12% TPWP addition were above 4, which was considered as the middle value in the 7‐point hedonic scale. Panelists commented that they perceived a bitter and astringent taste for the samples with 18% TPWP. It was thought that this was due to the bitter component in the furostanol saponin structure named TFI in tomato seeds (Katsumata et al. 2011).

**TABLE 8:** Sensory properties of biscuit samples.

Parameters	Biscuit samples			
	Control	TPW6	TPW12	TPW18
Color	4.54 ± 1.07^a^	4.60 ± 0.94^a^	4.73 ± 1.03^a^	4.50 ± 1.11^a^
Odor	4.98 ± 0.91^a^	4.73 ± 0.89^a^	4.58 ± 0.92^a^	4.08 ± 1.13^b^
Hardness	4.96 ± 1.15^ab^	5.02 ± 0.86^ab^	5.13 ± 0.73^a^	4.71 ± 0.87^b^
Taste	5.40 ± 1.03^a^	4.81 ± 0.96^b^	4.33 ± 1.06^c^	3.65 ± 1.41^d^
Chewiness	5.31 ± 0.90^a^	5.23 ± 0.93^ab^	5.27 ± 0.89^ab^	4.88 ± 1.08^b^
Overall Acceptability	5.21 ± 1.03^a^	4.77 ± 0.78^b^	4.31 ± 1.01^c^	3.73 ± 1.18^d^

*Note:* Different superscript letters in rows denote statistical differences (*p* < 0.05).

In the study of Nour et al. (2015), 6% and 10% dry tomato waste were used in bread production, and it was found that the samples had similar scores in terms of all sensory parameters (color, taste, aroma, texture, and overall acceptability). In another study (Bhat et al. 2017), cookies were produced by substituting wheat flour with tomato pomace at ratios of 5%, 10%, 15%, 20%, and 25%. It was reported that the sample with 5% tomato pomace had the highest score in overall acceptability, texture, taste, and color.

### 3.5 Integrated AHP‐TOPSIS Application

Table 9 shows the weights, ranking, and consistency ratios of the main criteria and sub‐criteria addressed in the evaluation of biscuit formulations as a result of AHP application.

**TABLE 9:** The weights of criteria and sub‐criteria and their consistency ratios.

Main‐criteria	Weights	Sub‐criteria	Weights	Results	Ranking	Consistency ratio
C1. Sensory properties	0.67760	C1.1. Taste	0.58651	0.39742	1	0.07305 acceptable
		C1.2. Color	0.10271	0.06960	5	
		C1.3. Chewiness	0.31078	0.21059	2	
C2. Functional properties	0.32240	C2.1. Total dietary fiber	0.11025	0.03554	7	0.03611 acceptable
		C2.2. Lycopene	0.38978	0.12566	3	
		C2.3. Antioxidant activity	0.37008	0.11931	4	
		C2.4. β‐Carotene	0.12989	0.04188	6	

As seen in Table 9, sensory properties appeared to be the most important main criterion, accounting for 67.760% of the total weight. This finding shows that sensory properties were very important in selection of the best biscuit formulation. Functional properties, on the other hand, were not as important as sensory properties, but they were still an important main criterion, accounting for 32.240% of the total weight. Among the sensory properties, taste had the highest weight with 39.742% and was considered the most important sub‐criterion. This was followed by chewiness with 21.059% and color with 6.960%. Among the functional properties, lycopene, antioxidant activity, β‐carotene, and total dietary fiber had weights of 12.566%, 11.931%, 4.188% and 3.554%, respectively. Lycopene and antioxidant activity were the most important sub‐criteria within functional properties. In summary, while taste was found to be the most important factor in the hierarchical structure, total dietary fiber was found to be the least important factor among the properties. It was observed that the calculated consistency ratios were within acceptable limits.

The criteria weights in Table 9 and the results of the experimental analysis were used as the decision matrix (Table 10) in the TOPSIS method.

**TABLE 10:** The decision matrix for the TOPSIS method.

Criteria →	C1. Sensory properties			C2. Functional properties			
Alternatives↓	C1.1. taste	C1.2. color	C1.3. chewiness	C2.1. total dietary fiber	C2.2. lycopene	C2.3. antioxidant activity	C2.4. beta carotene
A1. Control	5.40	4.54	5.31	3.33	0.00	2.08	0.00
A2. TPW6	4.81	4.60	5.23	5.54	3.68	10.42	12.85
A3. TPW12	4.33	4.73	5.27	7.37	6.57	28.23	14.75
A4. TPW18	3.65	4.50	4.88	9.94	8.28	35.47	16.75
Weights	0.39742	0.06960	0.21059	0.03554	0.12566	0.11931	0.04188

After the TOPSIS application, the calculated values of $S_{i}^{*},S_{i}^{-}$, and $C_{i}^{*}$ of each alternative are summarized in Table 11. The $C_{i}^{*}$ value given in Table 11 expresses the proximity of the alternatives to the ideal solution and it is interpreted that the alternatives with larger $C_{i}^{*}$ values perform better. Normalized $C_{i}^{*}$ was used to compare alternatives with each other. When the results of TOPSIS method for four alternative biscuits (Control, TPW6, TPW12, TPW18) given in Table 11 are examined, TPW12 alternative is evaluated as the best option for the normalized $C_{i}^{*}$ value according to the determined criteria. The TPW12 formulation was followed by TPW18 and TPW6 respectively, while the least preferred alternative was determined as control.

**TABLE 11:** The values of $S_{i}^{*}$, $S_{i}^{-}$ and $C_{i}^{*}$.

Alternatives	$S_{i}^{*}$	$S_{i}^{-}$	$C_{i}^{*}$	Normalized $C_{i}^{*}$	Ranking
A1. Control	0.13034	0.07622	0.36900	0.17420	4
A2. TPW6	0.08723	0.07213	0.45263	0.21367	3
A3. TPW12	0.05395	0.10744	0.66574	0.31428	1
A4. TPW18	0.07624	0.13034	0.63093	0.29785	2

In conclusion, according to the experts, sensory properties, especially taste, are of great importance in the selection of biscuit formulation. The functional properties of the biscuits are also important, but their influence on biscuit selection is not as effective as sensory properties. For example, although TPW18 outperformed the other alternatives in terms of functional properties, the high share of sensory properties, especially taste, in the decision‐making process caused it to be ranked second. TPW12, which performed better than TPW18 in terms of taste, ranked first.

## 4 Conclusion

Considerable production waste is generated during food processing. Transforming these wastes into high value‐added products is important to reduce environmental pollution and loss of biologically active components. TPW contains important bioactive food components such as dietary fibers, proteins, fats, minerals, phenolic compounds, and carotenoids. In this study, TPWP was added to the biscuit formulation at ratios of 6%, 12%, and 18% and the biscuits were analyzed in terms of chemical, physical, and sensory properties. It was determined that the addition of TPWP to the biscuit formulation enriched the samples in terms of protein, fat, ash, dietary fiber, lycopene, β‐carotene, mineral matter (P, K, Ca, Mg, Mn), and total phenolic matter. In the sensory analysis, it was found that the overall acceptability scores of the samples decreased with the increase in the TPWP addition ratio.

To determine the optimum biscuit formulation, the samples were examined in terms of sensory (taste, color, chewiness) and functional properties (total dietary fiber, lycopene, β‐carotene, antioxidant activity) using integrated AHP‐TOPSIS approach. According to the results of the AHP method, sensory properties constituted 67.760% of the total weight of the main criteria, while functional properties constituted 32.240% of the total weight. When the sub‐criteria were ranked in terms of weights, taste score ranked first with 39.742%, followed by chewiness score with 21.059%, and lycopene content with 12.566%. When biscuit formulations were ranked by TOPSIS method, it was determined that TPW12 formulation ranked first and therefore was chosen as the best alternative.

The present study aimed to contribute to the literature on waste management by using TPWP in biscuit formulation to reduce food waste. Furthermore, the integrated AHP‐TOPSIS approach used in the selection of the optimum biscuit formulation in this study might be an instructive method for similar multi‐criteria decision‐making studies in food engineering.

The evaluation of shelf‐life is an important aspect; however, it was not investigated in the current study. Future studies should focus on shelf‐life studies to investigate the stability of bioactive compounds in TPWP‐enriched products.

## Author Contributions

**Ezgi Ozgoren Capraz:** conceptualization (equal), data curation (equal), formal analysis (equal), investigation (equal), methodology (equal), writing – original draft (equal). **Ozan Capraz:** conceptualization (equal), data curation (equal), formal analysis (equal), methodology (equal), writing – original draft (equal). **Figen Turan:** conceptualization (equal), formal analysis (equal), investigation (equal). **Fatma Isik:** conceptualization (equal), data curation (equal), methodology (equal), supervision (lead), writing – original draft (equal).

## Conflicts of Interest

The authors declare no conflicts of interest.

## Data Availability

The data of the study are available from the corresponding author.
